# Cinnamaldehyde-Rich Cinnamon Extract Induces Cell Death in Colon Cancer Cell Lines HCT 116 and HT-29

**DOI:** 10.3390/ijms24098191

**Published:** 2023-05-03

**Authors:** Arti Nile, Jisoo Shin, Juhyun Shin, Gyun Seok Park, Suhyun Lee, Ji-Ho Lee, Kyung-Woo Lee, Beob Gyun Kim, Sung Gu Han, Ramesh Kumar Saini, Jae-Wook Oh

**Affiliations:** 1 Department of Stem Cell and Regenerative Biotechnology, KIT, Konkuk University, 120 Neungdong-ro, Gwangjn-gu, Seoul 05029, Republic of Korea; aartibmahajan@gmail.com (A.N.); 5shin96@naver.com (J.S.); junejhs@konkuk.ac.kr (J.S.); bhs2945@hanmail.net (G.S.P.); skiara1122@gmail.com (S.L.); 2 Department of Crop Science, Konkuk University, Seoul 05029, Republic of Korea; micai1@naver.com (J.-H.L.); saini1997@konkuk.ac.kr (R.K.S.); 3 Department of Animal Science and Technology, Konkuk University, Seoul 05029, Republic of Korea; kyungwoolee@konkuk.ac.kr (K.-W.L.); bgkim@konkuk.ac.kr (B.G.K.); 4 Department of Food Science and Biotechnology of Animal Resources, Konkuk University, Seoul 05029, Republic of Korea; hansg@konkuk.ac.kr

**Keywords:** cinnamon, cinnamaldehyde, colorectal cancer, apoptosis, cytotoxicity, MUDENG

## Abstract

Cinnamon is a natural spice with a wide range of pharmacological functions, including anti-microbial, antioxidant, and anti-tumor activities. The aim of this study is to investigate the effects of cinnamaldehyde-rich cinnamon extract (CRCE) on the colorectal cancer cell lines HCT 116 and HT-29. The gas chromatography mass spectrometry analysis of a lipophilic extract of cinnamon revealed the dominance of trans-cinnamaldehyde. Cells treated with CRCE (10–60 µg/mL) showed significantly decreased cell viability in a time- and dose-dependent manner. We also observed that cell proliferation and migration capacity were inhibited in CRCE-treated cells. In addition, a remarkable increase in the number of sub-G_1_-phase cells was observed with arrest at the G_2_ phase by CRCE treatment. CRCE also induced mitochondrial stress, and finally, CRCE treatment resulted in activation of apoptotic proteins Caspase-3, -9, and PARP and decreased levels of mu-2-related death-inducing gene protein expression with BH3-interacting domain death agonist (BID) activation.

## 1. Introduction

Colorectal cancer is one of the most common lethal cancers worldwide and was ranked as the third most common and lethal cancer in the U.S. population in 2022 [1]. Although extensive studies are underway to improve colorectal cancer treatments based on chemotherapy, monoclonal antibodies, and alternative therapies (e.g., macrobeads and anti-inflammatory drugs) [2], these treatments have been reported to have several side effects and cannot prevent recurrence [3]. To compensate for these flaws, it has been proposed that complementary and alternative medicines, including dietary supplements or herbal medicine, should be considered alongside conventional treatments [4]. 

Many plant-derived phytoextracts and natural dietary compounds such as phenolics, flavonoids, carotenoids, terpenoids, and peptides possess potential anti-cancer and cytotoxic properties [5]. Cinnamon, the dried bark obtained from several tree species of the genus *Cinnamomum*, is one of the most widely used aromatic condiments and flavoring additives worldwide [6]. It is also used traditionally in various cultures as a medicinal herb, and its positive effect on health has been scientifically recognized [7]. In vivo studies performed on rats show that ingested cinnamon extract does not have mutagenic or cytogenic effects, with minor nephrotoxicity and hepatotoxicity properties at high doses [8], underlying its overall safety for consumption. On the other hand, as reviewed [9], various extracts of cinnamon (i.e., water-soluble cinnamon extract, cinnamon bark essential oil, eugenol, cinnamaldehyde, and its derivatives) were reported to be effective in inducing apoptosis or inhibiting proliferation in diverse cancer types, suggesting their potential as a dietary complement in cancer treatment. Trans-cinnamaldehyde, the only natural form of cinnamaldehyde, is the principal component of cinnamon aromatic oil, for example, comprising up to 71.75% (*w*/*w*) of the oil from *Cinnamomum zelanicum* [10]. Cinnamaldehyde has been shown to affect apoptosis by the peroxisome proliferator-activated receptor and PI3K-Akt pathway in breast cancer [11], by ROS-mediated mitochondrial cell death in the leukemia cell line [12], by the CD95-mediated pathway in the hepatoma cell line [13], and by inducing oxidative stress in the melanoma cell line [14]. In colorectal cancer, cinnamaldehyde induces cell apoptosis in the SW480 [15], HT-29 [16], LoVo [15,16], and HCT 116 [15,17,18,19] cell lines. Cinnamaldehyde was also shown to affect cell invasion and adherence ability [15], sensitivity to chemotherapy [16], cell cycle [17,18], tubulin accumulation [17], and a cytoprotective effect at a sublethal concentrations via thioredoxin reductase (TrxR) inhibition [18] and oxidative stress response protein nuclear factor erythroid 2-related factor 2 (Nrf2) up-regulation [18,20]. Several derivatives of cinnamaldehyde, such as 2′-hydroxycinnamaldehyde or 5-fluoro-2-benzoyloxycinnamaldehyde, have been shown to have similar effects [17,18,19,21]. In addition to cinnamaldehydes, other active compounds in cinnamon extracts, such as eugenol [22], have been reported to have cytotoxic effects on cancer cells [23]. Moreover, as it has been previously described that several active components of cinnamon extract can act synergistically [24], cinnamon extracts might be more effective compared to pure cinnamaldehyde in treating cancer.

Based on the above considerations, the aim of the present study is to determine the anti-cancer effect of a cinnamon extract produced in a lab on colorectal cancer cell lines. In this study, the most abundant active compound of the cinnamon extract was determined to be cinnamaldehyde, and therefore the extract is identified as cinnamaldehyde-rich cinnamon extract (CRCE). We analyzed the effects of CRCE on colorectal cancer cell lines HCT116 and HT-29 in terms of cytotoxicity, colony formation ability, cell migration, cell cycle, mitochondrial potential change, and finally apoptosis-related protein expression.

## 2. Results

### 2.1. GC-MS Analysis of Cinnamon Extract

**Figure 1:**
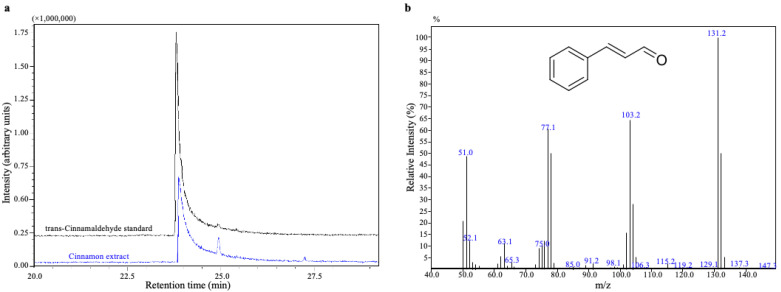
Gas chromatography (GC) analysis of cinnamon extract. (**a**) GC-total ion chromatograms (TICs) of trans-cinnamaldehyde standard and cinnamon extract. (**b**) GC-mass spectra (MS) of the trans-cinnamaldehyde detected from cinnamon extract.

We analyzed the active compound in the lipophilic cinnamon extract prepared as described in Materials and Methods. GC-MS analysis revealed the dominance of trans-cinnamaldehyde, which accounted for 52.2 g/100 g of the total extract (Figure 1a). GC-MS spectra analysis confirmed the identity of trans-cinnamaldehyde from cinnamon extract (Figure 1b). We conclude that the major active compound of the lipophilic cinnamon extract we isolated is trans-cinnamaldehyde, and therefore the extract is identified as cinnamaldehyde-rich cinnamon extract (CRCE). Moreover, palmitic (C16:0), stearic (C18:0), oleic (C18:1n9c), and linoleic (C18:2n6c) acids were identified as minor compounds.

### 2.2. CRCE Is Cytotoxic to HCT 116 and HT-29 Cells in a Dose- and Time-Dependent Manner

**Figure 2:**
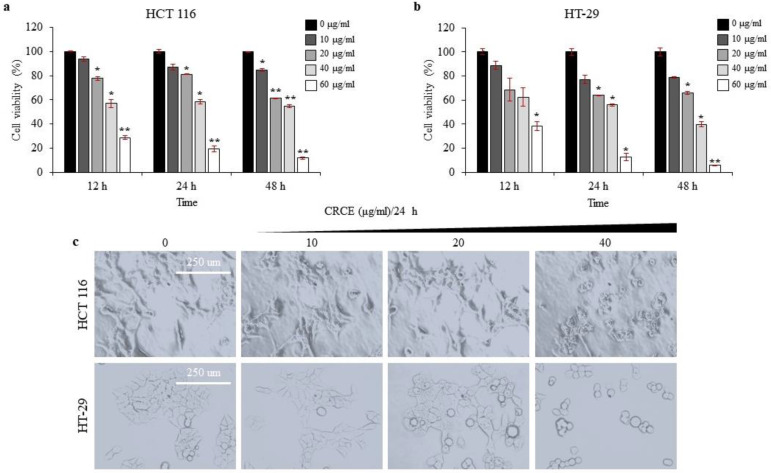
Evaluation of cytotoxic effect of CRCE in colorectal cancer cells. (**a**,**b**) WST-1 assays were used to evaluate cytotoxic effects of CRCE on HCT 116 and HT-29 cells in a dose- and time- dependent manner. Data in the graph are presented as mean ± SD of three biological replica. Two-tailed *t*-student test compared to control (* *p* < 0.01, ** *p* < 0.001). (**c**) Representative light microscopy image to analyze cellular morphological changes at 100× magnification in CRCE treatment for 24 h.

The CRCE effect on HCT 116 and HT-29 cells was assessed by WST-1 assay. As a result, CRCE treatment affected cell viability in dose- and time-dependent manners in both cell lines (Figure 2a,b). In HCT 116 cells, 10 µg/mL of CRCE treatment lowered cell viability at a statistically significant level upon treatment for 48 h (Figure 2a). In HT-29 cells, 10 µg/mL of CRCE treatment did affect cell viability in a time-dependent manner but not at a statistically significant level (Figure 2b), presumably due to the higher standard deviation. Overall, HT-29 cells responsivity to CRCE seems to be slower than that of HCT 116, as the IC_50_ values of CRCE for HCT 116 were 16.7, 13.5, and 18.9 µg/mL; for HT-29, they were 18.2, 16.3, and 9.3 µg/mL for 12, 24, and 48 h treatment, respectively. In addition to decreased cell viability, CRCE treatment resulted in the cell morphological change in HCT 116 and HT-29 cells (Figure 2c), which are hallmarks of apoptosis [25]. Based on these results, it is confirmed that CRCE is cytotoxic to the colorectal cancer cell lines HCT 116 and HT-29.

### 2.3. CRCE Inhibits Proliferation of HCT 116 and HT-29 Cells

**Figure 3:**
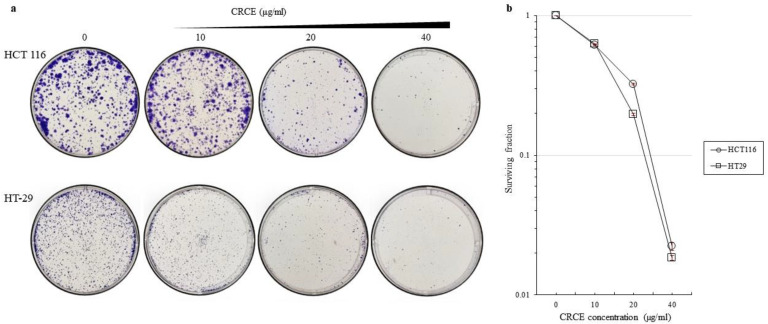
The effects of CRCE on colony formation in HCT 116 and HT-29 cells for 24 h. (**a**) Representative image of colony formation of HCT 116 and HT-29 cells treated with CRCE for 24 h before seeding. (**b**) Surviving fraction in three biological replicas. Data are presented as the mean ± SD.

Clonogenic assays were performed on both cell lines for two weeks after treatment with 0, 10, 20, or 40 µg/mL of CRCE for 24 h. As shown in Figure 3, both cell line clones decreased at a significant level upon CRCE treatment. Experiments were performed in three biological replicas, and a representative picture of the stained colonies is presented (Figure 3a). The plating efficiencies (PE) of HCT 116 and HT29 controls treated with DMSO were 1.503 and 1.9495, respectively. The surviving efficiency (SE) was calculated and plotted (Figure 3b). Both SEs of HCT 116 and HT-29 were significantly affected, even at the lowest CRCE concentration, as SE dropped to 0.61 and 0.62, respectively. The SE of cells treated with 40 µg/mL of CRCE for 24 h was as low as 0.02 and 0.18, respectively. This shows that CRCE greatly affected the cells’ ability to proliferate as colonies.

### 2.4. CRCE Inhibits Cell Migration of HT 116 and HT-29 Cells

**Figure 4:**
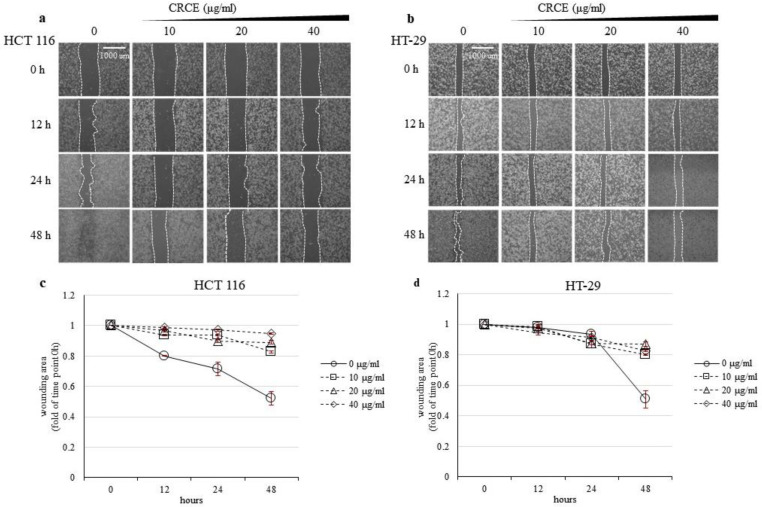
The effects of CRCE on cell migration in HCT 116 and HT-29 cells for 48 h. (**a**,**b**) Scratch wound-healing assay was used to evaluate the effect of CRCE on cell migration availability in HCT 116 and HT-29 cells. Representative images were captured using a light microscope at 10× magnification. (**c**,**d**). Graphs represent the wounding area change in HCT 116 and HT-29 cells upon CRCE treatment. The wounding areas were analyzed using ImageJ software. Data are presented as the mean ± SD of three biological replicas.

To investigate CRCE’s role in cell migration, we performed wound healing assays on CRCE-treated HCT 116 (Figure 4a,c) and HT-29 cells (Figure 4b,d). As shown in the microscope image (Figure 4a,b) and relative cell migration calculated by the % of the wounded area from the image (Figure 4c,d), HCT 116 and HT-29 cells treated with CRCE had a relative migration rate that decreased in the scratch wound healing assay. These results show that CRCE inhibited the migratory capabilities of both cell lines.

### 2.5. CRCE-Treatment-Regulated Cell Cycle of HT 116 and HT-29 Cells

**Figure 5:**
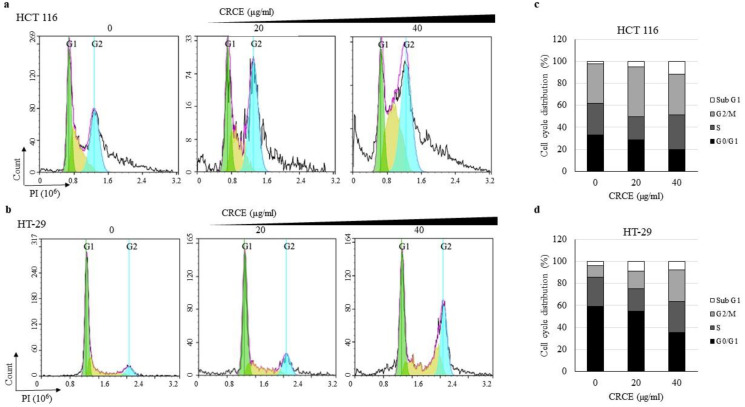
The effects of CRCE on cell cycles in HCT 116 and HT-29 cells for 24 h. (**a**,**b**) PI staining and analysis by flow cytometer were performed on cells treated with CRCE to assay cell cycle. The representative plot shows cells in G_0_/G_1_ phase (green), S phase (yellow), and G_2_/M phase (cyan). (**c**,**d**) Graphs show the percentages of cells in Sub G_1_, G_0_/G_1_, S, and G_2_/M phase. More than 10000 cells were analyzed for each treatment.

The effects of CRCE on the cell cycle were investigated in HCT 116 and HT-29 cells by flow cytometry analysis (Figure 5a,b). The total cell population and population of cells in each phase were calculated in % and presented in graphs (Figure 5c,d). As shown in the figure, CRCE treatment at 40 µg/mL increased the accumulation of cells in the sub-G_1_ phase by 11.5% compared to 2.23% in the control in HCT 116 cells and 7.8% compared to 3.7% in HT-29 cells. The G_0_/G_1_ phase population also decreased substantially in both cell lines, from 33.0% to 19.8% in HCT 116 cells and from 59.2% to 35.2% in HT-29 cells upon CRCE treatment in a dose dependent manner. Accordingly, the population of the cells that are in S + G_2_/M slightly increased from 64.6% to 68.6% and from 36.9% to 56.9% in HCT 116 and HT-29 cells. In HCT 116 cells, the sub-G_1_ population increase and G_1_ population decrease were correlated with treated CRCE concentration (Figure 5c). In HT-29 cells, the G_1_ population and G_2_/M population decrease were correlated with the treated CRCE concentration (Figure 5d). In short, we showed that upon CRCE treatment, dose-dependent G_1_ phase depletion with sub-G_1_ increase or G_2_/M phase arrest was observed in HCT 116 and HT-29 cells.

### 2.6. CRCE Treatment Induces Mitochondrial Stress in HCT 116 and HT-29 Cells

**Figure 6:**
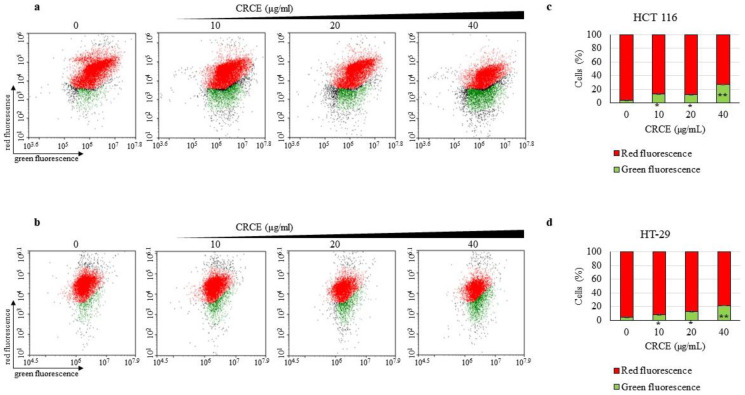
The effect of CRCE on the mitochondrial membrane potential (ΔΨm) of HCT 116 and HT-29 cells. (**a**,**b**) JC-1 assays was performed on HCT 117 and HT-29 cells treated with CRCE for 24 h. The dot plots represent the JC-1 dyed cells with healthy mitochondria (red fluorescence) or depolarized mitochondria (green fluorescence). (**c**,**d**) Bar graph indicating the percentages of polarized and depolarized cells based on JC-1. Data are expressed as mean ± SD from three independent experiments. Two-tailed *t*-student test compared to control (* *p* < 0.05, ** *p* < 0.005).

We investigated the CRCE effect on mitochondrial stress by analyzing the change in mitochondrial transmembrane potential (Δψm). In cells with healthy mitochondria, JC-1 enters the mitochondria and forms J-aggregates with red fluorescence, while in cells with stressed mitochondria (low Δψm), monomeric green fluorescent JC-1 remains in the cytoplasm. Dot plots (Figure 6a,b) represent the cells with lower-right green fluorescence and the upper left with red fluorescence. To improve visualization, bar graphs of the red versus green cell populations were plotted (Figure 6c,d). As shown in Figure 6, CRCE significantly depolarized the mitochondrial membrane potential of HCT 116 and HT-29 cells after 24 h of treatment.

### 2.7. CRCE Induces Cell Apoptosis of HCT 116 and HT-29 Cells

**Figure 7:**
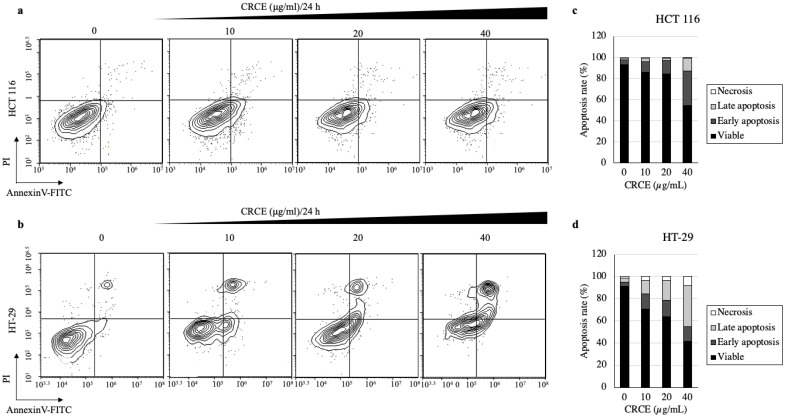
The effect of CRCE on cell apoptosis in HCT 116 and HT-29 cells. (**a**,**b**) PI and FITC-Annexin V co-staining was used to identify cell death upon CRCE treatment for 24 h (**c**,**d**) Graphs indicating the percentages of apoptotic cells. Experiments were performed in triplicate, and the results of one representative experiment is shown. PI: propidium iodide, FITC: fluorescein isothiocyanate.

The analysis of apoptosis in colorectal cell lines upon CRCE treatment was performed with the Annexin V/PI double staining kit (Figure 7a,b). Based on the data of more than 10,000 cells, percentages of cells at specific stages (viable, early or late apoptosis, and necrosis) were presented in graphs (Figure 7c,d). Upon CRCE treatment, viable cells decreased from 93.54% to 54.21% or from 91.27% to 41.8% in HCT 119 or HT-29, respectively, in dose-dependent manners. Accordingly, cells in early or late apoptosis and necrosis increased upon CRCE treatment in a time-dependent manner. In HCT 116, most of the increase in apoptotic cell population was due to an increase in the early apoptotic cell population, whereas in HT-29, it was due to an increase in the late apoptotic cell population, with a small but substantial increase in necrotic cells. 

### 2.8. CRCE Affected Expression of Apoptotic Pathway Related Proteins

**Figure 8:**
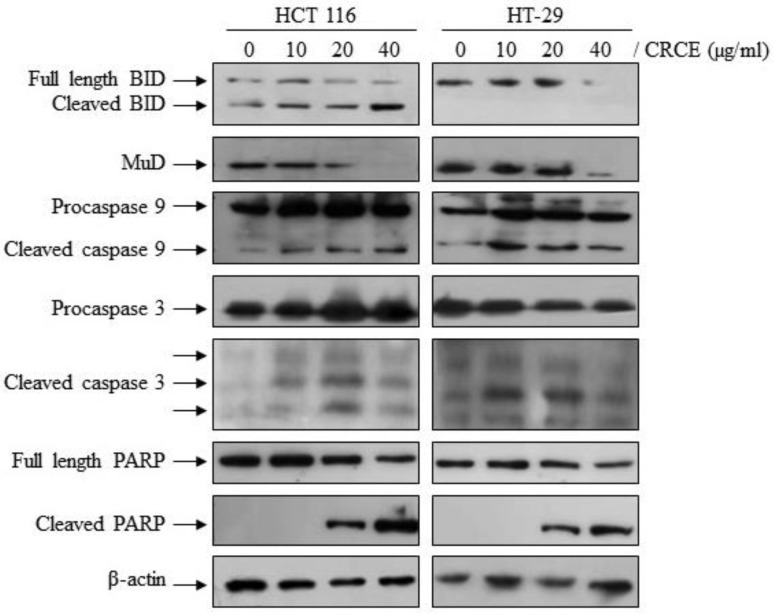
The effect of CRCE on apoptosis-related protein in HCT 116 and HT-29 cells. Representative western blots of BID, MuD, Caspase-9, Caspase-3, PARP, and β-actin upon cells treated with CRCE for 24 h.

To investigate the mechanism of CRCE-induced apoptosis at the molecular level, we examined its effect on the cascade of caspase-mediated signaling that is crucial in initiating cell death pathways. Protein levels and a cleaved form of caspase-3 and caspase-9 were observed by western blot analysis in HCT 116 and HT-29 cells treated with CRCE. As shown in Figure 8, CRCE induced the activation of procaspase-9 into cleaved caspase-9 and of procaspase-3 into the active cleaved caspase-3. Bid and PARP, two substrates of activated caspases, were also shown to be cleaved or downregulated upon CRCE treatment. Interestingly, MuD, a novel gene that plays an important role in cell death in various tissues [26], has been shown to be downregulated by CRCE treatment. These observations strongly indicate CRCE-induced apoptosis in HCT 116 and HT-29 cells via activation of the caspase cascade.

## 3. Discussion

In this study, we produced CRCE from cinnamon quills that are composed of 52.5% cinnamaldehyde. We further showed that CRCE affected cell proliferation, migration, cell cycles, mitochondrial potential, and apoptosis in the colon cancer cell lines HCT 116 and HT-29.

As mentioned in the introduction, cinnamon extracts’ and active compounds’ effects on colon cancer cells have been studied. As cinnamaldehyde is the major compound in cinnamon extracts, their effect on cancer cells is usually thought to be induced by this chemical. Indeed, HCT 116 treated with pure cinnamaldehyde showed cytotoxicity at concentrations starting at 20 µg/mL for 24 h [15], and HT-29 IC50 for cinnamaldehyde was around 9.12 µg/mL [16]. However, extracts isolated with different methods consist of a mixture of chemicals, and while cinnamaldehyde is the main compound, the effects of extracts might differ or be synergistic. For example, it was previously shown that a cinnamon extract with 92.8% cinnamaldehyde had cytotoxic properties in HCT 116 cells and affected mitochondrial health at concentrations as low as 0.4 µg/mL for 12 h [27]. In this study, CRCE, which consists of around 52.2% cinnamaldehyde, was efficient in both HCT 116 and HT-29 cells at concentrations as low as 10 µg/mL; the IC_50_ was 13.5 and 16.3, respectively. Therefore, based on a previous study, CRCE was slightly more cytotoxic than pure cinnamaldehyde in both HCT 116 and HT-29 cell lines. This might imply that in treatment, extracts of cinnamon might be more effective compared to pure cinnamaldehyde. The other major bioactive compounds in cinnamon are A-type proanthocyanidin polymers, cinnamic acid, and coumarin [28]. Further studies should be performed to analyze both the independent and synergistic effects of those compounds.

Exposure to phytochemicals through diet or drug administration can halt or delay carcinogenic processes. Phytochemicals are thought to play a role in reducing the incidence of colon cancer [29]. Polyphenols, such as flavonoids, phenolic acids, and sesquiterpenoids, are known for their ability to induce apoptosis in colon cancer cells [30,31]. In addition, high levels of ROS contribute to the progression of a variety of disorders and multiple cancers, including colorectal cancer [32]. It is well known that the main role of antioxidants is to protect them from damage caused by ROS, reducing their risk of cancer. Cinnamaldehyde decreases the expression of nitric oxide, interleukin (IL)-1β, IL-6, and tumor necrosis factor (TNF)-α in lipopolysaccharide (LPS)-activated BV2 microglial cells [33] and has been reported to be a reducing factor in myoblasts [34]. In the rat model, orally injected cinnamaldehyde had a beneficial effect on the gut microbiome [35], therefore showing that cinnamaldehyde, as well as other antioxidants, are considered as healthy dietary supplements. Interestingly, in the colon cancer cell lines HCT 116, HT-29 (that was studied here), and immortalized epithelial human colon cells of fetal origin (FHC), it was shown that cinnamaldehyde has a protective effect on oxidative stress by up-regulating the anti-inflammatory protein Nrf2 [19]. In this study, we observed that cinnamaldehyde-enriched CRCE induced HCT 116 and HT-29 cell mitochondrial stress, which correlates with oxidative stress by increasing ROS [36]. The discrepancy between these results might be because CRCE consists of several other compounds besides cinnamaldehyde, and/or cinnamaldehyde has both anti- and pro-oxidant activity. Indeed, it was previously shown that, in contrast with healthy cells, antioxidative agents are proposed to increase the effect of chemotherapeutic agents [37] and trigger apoptosis in cancer cells [38]. The possibility of controlling ROS to trigger cancer cell apoptosis while protecting adjacent healthy cells with antioxidants was recently reviewed [39].

In this study, CRCE induced cell cycle arrest in the G_2_/M phase, which is similar to the results of previous studies using cinnamon extract in various human cancer cell lines [40,41]. The sub-G_1_ phase increase is characteristic and indicative of apoptotic bodies [42]. G_2_/M phase arrest is due to the failure to pass checkpoints that are usually due to DNA damage [43]. The central machinery that promotes cell cycle progression is regulated by Cdk [44], which in turn is regulated by cell-cycle-specific interactions with cyclins and Cdk inhibitors. P53 is a protein encoded by the *TP53* gene that plays a role in regulation or progression via the cell cycle, apoptosis, and genomic stability. Fischer et al. [45] found that the p53-p21-DREAM-CDE/CHR pathway involves 210 genes that regulate the G_2_/M cell cycle in HCT 116 cells. The downregulation of most of these genes appears to be the primary mechanism by which p53 arrests the G_2_/M cell cycle. This may represent G_2_/M phase arrest in experiments with HCT 116 and HT-29 cells; however, more studies are needed to confirm the involvement of p53. Moreover, we have shown by clonogenic assay that CRCE decreased the proliferation ability of viable cells. This also suggests that the proliferation ability might have been impaired by DNA damage, while we cannot rule out the possibility that the cytotoxic effect of CRCE impaired the seeded cells’ viability [46]. We assume that the activity of CRCE is exerted via different pathways, which still need to be explored, as both the increase in sub-G_1_ and Annexin V/PI assays confirmed that CRCE induces apoptosis and that cell cycle arrest is an intermediate step prior to apoptotic death.

Apoptosis is most clearly characterized by cytoplasmic and nuclear condensation, followed by nucleosomes, DNA breaks between membrane specks, and finally cellular damage [47]. In most cases, anti-cancer therapy ultimately leads to caspase activation, which plays an important role in apoptosis. We investigated the dose-dependent activation of caspases by western blot analysis using anti-caspase-3 and anti-caspase-9 Abs. According to the results here, CRCE induced the activation of caspase-3 and caspase-9 in both cell lines. Following caspase-3 activation, the cleavage of the active form of PARP, which represents cell survival, is an important step in the completion of the apoptotic program. Western blot analysis showed that CRCE treatment resulted in PARP cleavage in these cells in a dose-dependent manner. The mu-2-related, death-inducing gene (MUDENG, *MuD*) was reported to be involved in the apoptotic pathway in various cancer cell lines [48,49,50]. Subsequent studies demonstrated the cleavage of MUDENG by active caspase-3 during TRAIL-induced apoptotic signaling [51] and the subsequent activation of the anti-apoptotic function of MUDENG near the BH3-interacting domain death agonist (Bid) and B-cell lymphoma 2 (Bcl-2) junction [26]. Previous studies have shown that Bid proteins inhibit MuD expression and that apoptotic response by TRAIL, which cleaves Bid into active Bid (tBid), is inhibited by MuD in a Bid-protein-dependent manner [26]. Here, we have found that the expression of MuD was significantly reduced and that the Bid protein was cleaved by CRCE treatment. As previously described by Fulda and Devatin [52], the use of the extrinsic and intrinsic mechanisms of apoptosis can be inferred from the cross-talk between the two apoptotic pathways. Thus, we suggest that CRCE treatment induced an extrinsic apoptotic pathway, which results in tBid migration to the mitochondria and activates the endogenous apoptotic pathway that can be shown by MuD degradation, activation of caspase-3 and caspase-9, and cleavage of PARP.

## 4. Materials and Methods

### 4.1. Plant Material and Lipophilic CRCE Extraction

Authentic cinnamon quills (obtained from the inner bark of *Cinnamomum verum J. Presl*) were procured from a commercial supplier (Foreign Mart, Itaewon, Seoul, Republic of Korea). The cinnamon quills were powdered using a domestic food processor and sieved through a 500 mm mesh. A total of 20 g of cinnamon quill powder was homogenized with 150 mL of isopropanol/cyclohexane (3:2, *v*/*v*) in a 250 mL conical flask. After, extraction was performed using a bath sonicator JAC-2010 at 300 W and 60 Hz for 10 min to enable sufficient disintegration and complete extraction. The obtained extract was vacuum-filtered, and the pellets were extracted again with 50 mL of cyclohexane. The filtrate containing lipophilic compounds was pooled, transferred to a 500 mL separating funnel, and partitioned with 100 mL of 1% NaCl. The upper cyclohexane fraction containing lipophilic compounds was recovered, transferred to a 500 mL round bottom flask, and vacuum-dried in a rotary evaporator at 35 °C. The weight of the lipophilic extract was measured gravimetrically and was dissolved in dimethyl sulfoxide (DMSO; 100 mg/mL of extract) for cell treatment or in hexane for gas chromatography mass spectrometry (GC-MS) analysis [25]. All organic solvents used for extraction were of liquid chromatography (LC)-MS grade and were obtained from J.T. Baker^®^ (Avantor Performance Materials Korea Ltd.), Suwon, Republic of Korea.

### 4.2. GC-MS Analysis of Cinnamon Extract

A fraction of the prepared cinnamon extract was dissolved in hexane (0.05 mg/mL) and analyzed using QP2010 SE GC-MS (Shimadzu, Tokyo, Japan). The analytical conditions are listed in Table 1. Quantitative analysis was performed using a calibration curve (0.1–0.01 mg/mL) with a trans-cinnamaldehyde standard (Merck, Rahway, NJ, USA) as per the protocol described by Wang et al. [53].

### 4.3. Cell Lines and Culture Condition

The human colorectal cancer cell lines HCT 116 and HT-29 were obtained from the Korean Cell Line Bank (Seoul, Republic of Korea) and determined to be mycoplasma-free based on the Biomax Mycoplasma PCR Analysis Kit (Biomax Inc., Daejeon, Republic of Korea). Cells were maintained in McCoy’s 5A medium supplemented with 10% heat-inactivated (*v*/*v*) fetal bovine serum (FBS), 2 mM L-glutamine, 10,000 units/mL penicillin, 10,000 of µg/mL streptomycin, and 1 mM of sodium pyruvate. All mediums, supplemental serum, and antibiotics were purchased from Welgene (Gyeongsan, Republic of Korea).

### 4.4. Cell Viability Assay

The viability of HCT 116 and HT-29 cells treated with CRCE (0–60 μg/mL) for 12, 24, and 48 h was assayed by the WST-1 assay kit (Dogen, Seoul, Republic of Korea). Briefly, the HCT 116 and HT-29 cells were seeded in a clear-bottom 96-well plate at a density of 1 × 10^3^ cells per well and grown overnight before the replacement of a serum-free medium with CRCE or DMSO as the control and incubation for 12, 24, or 48 h. After treatment, 10 μL of WST-1 solution was added per well, and plates were incubated for an additional hour. Finally, a SpectraMaxM2 microplate reader (Molecular Devices, San Diego, CA, USA) was used to measure plates’ absorbance at 450 nm, with 600 nm as the reference wavelength as per the manufacturers’ protocol.

### 4.5. Colony Formation Assay

HCT 116 and HT-29 cells were seeded in 6-well plates at a density of 2.5 × 10^5^ cells per well and grown overnight. After treatment with 10, 20, or 40 µg/mL CRCE or DMSO as a control for 24 h, cells were trypsinized, counted, and 1000 cells per well were re-seeded in 6-well plates. Cells were further cultivated for 7 days with the medium refreshed every other day to allow colonies to grow. Obtained colonies were washed with PBS, fixed with methanol for 10 min at room temperature (RT, 20 to 25 °C), washed with PBS, and stained with a 0.5% crystal violet solution (Junsei Chemicals, Tokyo, Japan). After washing in water and adequate drying, colonies on the six-well plates were counted, and the seeding efficiency (SE) and surviving fraction (SF) were evaluated with the previously described method [54].

### 4.6. Wound Healing Assay

HCT 116 and HT-29 cells were seeded in 6-well plates at a density of 5 × 10^4^ cells per well and cultured for 24 h to allow cell adhesion and the formation of a confluent monolayer. A straight-line scratch was then made using a sterile pipette tip to leave a scratch width of approximately 0.5 mm, which was then washed with PBS to remove debris. The cells were then treated with 10, 20, or 40 µg/mL of CRCE or DMSO as the control. After treatment, the wounding area was measured at 0, 12, 24, and 48 h after treatment using the Zeiss Axiovert 200 M inverted microscope (Carl Zeiss, Oberkochen, Germany) and computed as graphs, and the rate of cell migration were calculated as described previously [55].

### 4.7. Cell Cycle Analysis

To perform cell cycle analysis, HCT 116 and HT-29 cells were seeded in 6-well plates at a density of 2.5 × 10^5^ cells per well. After overnight incubation, cells were treated with 10, 20, or 40 μg/mL of CRCE or DMSO as the control for 24 h. After treatment, cells were collected via trypsinization and centrifuged at 850× *g* for 5 min, washed with PBS, and fixed with 70% ethanol *in PBS* at 4 °C for 1 h. Subsequently, cells were washed twice with PBS, and 50 μL of 100 μg/mL stock of DNase-free RNase was added *to digest* RNA. After RNase treatment, cells were stained with 50 µg/mL propidium iodide (PI) (BD Life Science, San Diego, CA, USA) for 30 min at RT. The PI intensity was measured using the NovoCyte 1000 benchtop flow cytometer (ACEA Biosciences, San Diego, CA, USA).

### 4.8. Mitochondrial Membrane Potential Analysis

HCT 116 and HT-29 cells were seeded in 6-well plates at a density of 2.5 × 10^5^ cells per well and incubated overnight. After that, cells were treated with 10, 20, or 40 µg/mL of CRCE or DMSO for 24 h before harvesting by trypsin. Cells were resuspended in 1× assay buffer and stained with JC-1 from the Mito Probe JC-1 Assay Kit (Enzo Life Sciences, New York, USA) for 30 min at RT. The cells were then washed and resuspended in PBS for flow cytometric analysis using the NovoCyte 1000 benchtop flow cytometer (ACEA Biosciences, San Diego, CA, USA) to detect red/green fluorescence changes in cells mitochondrion.

### 4.9. Apoptosis Assay

HCT 116 and HT-29 cells were seeded in 6-well plates at a density of 2.5 × 10^5^ cells per well and incubated overnight. Cells were treated with 10, 20, or 40 μg/mL of CRCE or DMSO as the control for 48 h. After treatment, cells were harvested, washed twice with cold PBS, and stained with the Annexin V-FITC/7-AAD apoptosis detection kit (Sino Biological, Beijing, China). The apoptotic states in each cell group were detected using the NovoCyte 1000 benchtop flow cytometer (ACEA Biosciences, San Diego, CA, USA).

### 4.10. Western Blot Analysis

Differential protein expression after CRCE treatment in HCT 116 and HT-29 cells was observed by western blot. Cells were seeded in 6-well plates at a density of 2.5 × 10^5^ cells per well and incubated overnight. Plated cells were treated with DMSO, 10, 20, or 40 µg/mL of CRCE for 24 h before harvest and lysis with RIPA lysis buffer (Thermo Fisher Scientific, Waltham, MA, USA). Cellular protein concentrations were determined by the Bio-Rad Protein Assay Kit (Bio-Rad, Hercules, CA, USA). A total of 50 μg of cellular proteins from each treatment was subjected to SDS-PAGE and transferred to polyvinylidene difluoride membranes by electrophoresis. Membranes were blocked with 5% (*w*/*v*) skim milk for 1 h at RT and incubated with primary antibodies diluted in 5% skim milk at the following dilutions: β-actin (1:5000), Caspase-3 (1:1000), Caspase-9 (1:1000), Bid (1:1000), Bcl-2 (1:1000), PARP (1:1000), and MUDENG (1:5) at 4 °C overnight. Caspase-3, -9, and PARP antibodies were purchased from Cell Signaling Technology (Danvers, MA, USA). Bid antibodies were purchased from Santa Cruz Biotechnology (Dallas, TX, USA). MUDENG antibodies were produced in this lab [56]. Thereafter, the membranes were rinsed with TBS-T five times for 5 min each and incubated with the appropriate horseradish peroxidase-conjugated secondary antibodies purchased from Jackson Immuno Research (West Grove, PA, USA) at RT for 2 h. After washing membranes in TBS-T five times for 5 min each, immunoreaction was detected by applying the enhanced chemiluminescence detection kit solution (Amersham Pharmacia, Uppsala, Sweden) on washed membranes and developing on X-ray film (AGFA, Mortsel, Belgium).

## 5. Conclusions

We have found that CRCE treatment induces a reduction of mitochondrial Δψm, which is one of the main events in the apoptosis induced by chemotherapeutic drugs [57]. We also observed cell cycle arrests and a decrease in proliferation that might be linked to DNA damage. In addition, we found cell motility decreased upon CRCE treatment. Finally, we observed the activation/degradation of extrinsic and intrinsic apoptosis-related proteins. Other studies have shown that the antitumor effects of CRCE appear to be mediated by multiple mechanisms [58,59].

## Figures and Tables

**Table 1:** GC-MS Parameters used for the analysis of sterols.

Gas Chromatograph Parameters			
Column over temperature	70 °C		
Injection Temperature	275 °C		
Injection mode	Split		
Carrier gas	Helium		
Flow control mode	Liner velocity		
Pressure	62.1 kPa		
Total Flow	9.0 mL/min		
Column Flow	1.0 mL/min		
Liner velocity	36.7 cm/sec		
Purge flow	3.0 mL/min		
Column	DB-5ms (30 m, 0. 25 μm film thickness, 0.25 mm ID; Agilent 7890B, Agilent Technologies Canada, Inc., Mississauga, ON, Canada		
Column over temperature	Rate (°C/min)	Final temperature (°C)	Hold time (min)
	-	70	10
	5	300	20
Total program time	76.0 min		
**Mass spectrometer parameters**			
Ion source temperature	250 °C		
Interface temperature	280 °C		
Solvent cut time	12 min		
Acquiring mode	Scan		
Event time	30 s		
Detector voltage	1.5 kv		
Scan speed	2500		
Start *m*/*z*	50.00		
End *m*/*z*	650.00		
